# Federated spatio-temporal graph neural network for privacy-preserving vehicle trajectory prediction in autonomous driving

**DOI:** 10.3389/frai.2026.1856630

**Published:** 2026-08-06

**Authors:** Aditi Joshi, Amrutha P, Vaidehi Prajapati, Sudha Anbalagan, Suganeshwari G

**Affiliations:** 1 School of Computer Science and Engineering, Vellore Institute of Technology, Chennai, Tamil Nadu, India; 2 Centre for Smart Grid Technologies, School of Computer Science and Engineering, Vellore Institute of Technology, Chennai, Tamil Nadu, India

**Keywords:** autonomous driving, federated learning, graph neural networks, privacy preserving, spatio-temporal

## Abstract

Accurate vehicle trajectory prediction plays a vital role in autonomous driving and intelligent transport systems. Deep learning models like LSTM, CNN, GNN, etc., have shown remarkable performance but often operate in a centralized setting, aggregating raw trajectory data at the server. Furthermore, the majority of models focus on either spatial or temporal features alone, but overlook the information that can be obtained by combining spatio-temporal features. This leads to major privacy concerns, issues with centralized data, and scalability problems. To overcome these challenges, we introduce a Federated Spatio-Temporal Synchronous Dynamic Graph Neural Network (Federated STSDGNN) framework for privacy-preserving and adaptive trajectory prediction. Using the highD dataset, trajectories are segmented into spatiotemporal sequences and represented as dynamic interaction graphs. Each client (vehicle or roadside unit) locally trains an STSDGNN consisting of a pre-processing module, a spatial-temporal synchronization module (GCN/GAT with GRU) and a prediction module (CNN with MLP). Clients send only model updates, which are aggregated by the server using a federated learning algorithm. This design improves privacy, achieves approximately 23.5% lower RMSE compared to the centralized STSDGNN baseline in the conducted experiments, and has the potential to support future privacy-preserving V2X (Vehicle-to-Everything) applications.

## 1 Introduction

Autonomous driving has been a revolutionary technology in recent years, providing services that improve everyday life, including safety warnings, motion planning, and intelligent driving decision-making (Garikapati and Shetiya, 2024). The adoption of autonomous vehicles (AVs) has been a matter of safety concerns. In recent times, many accidents caused by self-driving cars have been reported, emphasizing the vital need for precise trajectory prediction to improve vehicle safety. By forecasting how nearby vehicles are likely to move, autonomous systems can predict potential collisions and make timely decisions that can reduce traffic accidents and improve road safety. Thus, it is critical to improve the accuracy of vehicle trajectory prediction to continue to improve autonomous driving technologies (Lee and Curran, 2025).

Precise trajectory prediction involves massive data collection and intricate model training. The conventional centralized method, which collects raw data from vehicles in bulk to train the model, suffers from huge privacy, communication cost, and scalability issues (Leon and Gavrilescu, 2021). Federated Learning (FL) has proven to be a successful distributed machine learning paradigm to overcome these issues. In FL, local devices or vehicles learn local models from their own data and send only model updates, never raw data, to a central server. The server combines the updates to update a global model, which is subsequently sent to all members. This cycle promotes collaborative learning while preserving privacy and reducing communication overhead. Federated learning (FL) was first introduced in mobile phones, where each device contributed to a global model, but sensitive user data was not shared (Konečný et al., 2017; Yang et al., 2019; Li T. et al., 2020).

Current autonomous driving models tend not to make the most of the rich spatio-temporal interplay that is characteristic of traffic situations. Traditional trajectory prediction approaches, for example, physics-based models, maneuver-based models, and RNN-based methods such as GRUs and LSTMs, usually address vehicles in isolation and make only short-term predictions. Although interaction-aware techniques and attention mechanisms have enhanced prediction by taking inter-vehicle impacts into account, most methods continue to address spatial and temporal relations independently (Xu et al., 2025). In the same manner, graph-based models depict vehicles as nodes and relations as edges, but most current methods design static graphs with constant edges, which restrict them in portraying highly dynamic driving scenes (Ren and Zhang, 2024).

The paper does not propose a new graph neural network architecture or a new federated optimization algorithm. Instead, it presents a system-oriented, privacy-preserving trajectory prediction framework for decentralized autonomous driving. The proposed framework integrates the STSDGNN architecture into a FedAvg-based federated learning environment (Li X. et al., 2020), enabling collaborative model training without sharing raw trajectory data. This FL architecture is combined with spatio-temporal graph-based neural networks to predict vehicle trajectories. By encoding vehicles as nodes in temporal graphs and describing how they interact at different times, our method learns intricate spatio-temporal dependencies while maintaining privacy (Shen et al., 2023). Scalability over large AV networks is ensured by communication-efficient aggregation techniques. The suggested framework overcomes the drawbacks of static graph modeling and independent spatial-temporal extraction, and presents an efficient, privacy-enforcing, and scalable framework for precise trajectory prediction in real-world autonomous driving scenarios.

### 1.1 Literature review

Zhang et al. (2021) performed one of the first case studies, introducing an end-to-end FL framework for vehicle systems in real time. The approach was based on distributed model training with parameter aggregation among vehicles, tested on driving-related perception tasks. The results exhibited feasibility, but communication overhead and scalability were significant limitations. Chellapandi et al. (2023) followed through with this process by having a comprehensive overview of FL in CAVs, classifying approaches in perception, prediction, and communication tasks. Although the datasets were borrowed from existing benchmarks, the analysis highlighted open issues such as non-IID data, latency, and deployment.

Based on these works, Cui et al. (2025) suggested FLAV, a novel framework that combines parameter filtering and multi-chain parallel aggregation to achieve greater efficiency and privacy. They used MNIST and CIFAR-10 datasets for their approach, which achieved faster convergence than FedAVG, though real-world AV datasets were not tested. Nguyen et al. (2022) proposed a novel deep federated learning (DFL) infrastructure using FADNet that allows vehicles to support peer-to-peer training without the use of a central server. Although their results using autonomous driving datasets for the navigation mode showed quite a bit of robustness, the heterogeneity of the client data was a bottleneck.

At the perception level, Jallepalli et al. (2021) developed a federated learning (FL) framework for object detection using YOLO models with the testing completed from automotive vision datasets. The system provided an accuracy level similar to that of centralized training while safeguarding raw data, although it required large-scale validation. Ali et al. (2025a) conducted a survey on privacy, performance, and scalability challenges, reviewing methods such as gradient compression and differential privacy. They stated that while benchmark datasets (e.g., ImageNet, KITTI) are very common to use, real-world AV datasets are currently under-represented. This was complemented by Wang (2024), who covered the methodological problems of FL security, such as defenses against gradient leakage and model poisoning, with emphasis on experimental frameworks rather than data-driven analysis.

Ali et al. (2025b) broadened the scope of FL further by investigating its applicability to mobility, safety, and sustainability using a conceptual research approach that integrated existing case studies and simulation-based analysis. Li et al. (2022) examined privacy-preserving FL by the use of encryption techniques in conjunction with federated aggregation to protect the trajectory information of autonomous vehicles. They assessed their approach using simulated trajectory data, reporting improved privacy-accuracy trade-offs among their added encryption techniques. Xiang (2024) authored another survey paper, mapping current FL methods across perception and communications, emphasizing methodological gaps such as a lack of adequate end-to-end management of non-IID data in large-scale vehicular environments.

From a design perspective, Wang et al. (2023) proposed an FDL architecture for AV perception, emphasizing sensor fusion techniques and testing across multi-sensor data to validate. Their result showed that distributed models can achieve the same accuracy as centralized learning. Acar and Sterling (2023) considered reliability by proposing infrastructure-enabled FL, in which roadside units participated in aggregation to stabilize training and communication. Their simulation-based assessment discovered enhanced reliability but created dependency risks. Lastly, Lindskog-Munz ing and Prehofer (2025) explored the overall car ecosystem with conceptual methods for adaptive aggregation and communication-efficient FL, pointing out the absence of standardized datasets and evaluation protocols in vehicular domains.

Sadid and Antoniou (2024) proposed a dynamic spatio-temporal graph neural network for autonomous vehicle path prediction with an innovative adjacency matrix capturing inter-vehicle relationships based on distance and strategic position. Their model achieved a 34% improvement in trajectory prediction error over the baseline on a 5-s horizon and a 28% improvement in mean error. Sheng et al. (2022) proposed the Graph-Based Spatial-Temporal Convolutional Network (GSTCN), an integration of GCNs for spatial relationships and CNNs for temporal dependency, and GRUs for trajectory prediction on NGSIM's I-80 and US-101 datasets. GSTCN enhanced accuracy by 22.4% and had more efficient inference and a smaller model size.

The STS-DGNN model Li et al. (2023) proposed a dynamic graph neural network that synchronously models spatial-temporal dependencies, tested on highD, EWAP, and UCY datasets, with up to 32.3% ADE and 36.3% FDE improvement over T-GNN and 19.3% ADE and 29.7% FDE outperforming AgentFormer. This fulfilled the shortage in high-order spatiotemporal modeling but commented on multimodal motion and heterogeneous road user treatment. Likewise, the STGNN architecture (Chen et al., 2021) incorporated stacked GRUs with non-local processing for temporal encoding and a GNN block to model interactions explicitly. Evaluated on a self-designed unstructured dataset (≈4,000 samples) and Argoverse (333k sequences), it lowered errors to as much as 28% ADE and 23% FDE over baselines such as SocialLSTM, SocialGAN, SpaGNN, and CVM, solving for the absence of explicit interaction and temporal dependency modeling.

Another improvement, the Spatial-Temporal Graph Attention Transformer (STGATr) (Zhang et al., 2022), utilized graph attention for interaction modeling and transformers for long-range dependencies. On Argoverse, it lowered errors by 21% ADE and 18% FDE, breaking the isolation between spatial and temporal modeling but still not having multimodal prediction. Another work Singh and Srivastava (2022) developed a multi-scale GNN with temporal convolutions and an LSTM encoder-decoder, tested on Apolloscape, Lyft Level-5, and Argoverse, with ADE/FDE of 1.49/1.89, 1.57/1.25, and 0.53/0.87, respectively, decreasing errors by 50%–70% against baselines, especially useful for sparse datasets. Lastly, the IA-STDGNN (Wang et al., 2025) combined feature extraction, normalization, dynamic GCNs with single-lane and multi-lane mechanisms, spatiotemporal fusion, and multimodal prediction. On NGSIM (US-101, I-80) and HighD, it attained 0.80 m RMSE on NGSIM (error decrease of 13.98%–48.72%) and 0.19 m RMSE on HighD (38.71% improvement), with outstanding performance particularly for long-term prediction.

The STA-LSTM model (Lin et al., 2022) integrates LSTMs with spatial-temporal attention for enhanced accuracy and explainability compared to physics, maneuver, and interaction-based techniques. With the NGSIM dataset (US-101 and I-80), it attained an RMSE of 0.5615 at the 5th step, minimizing error by 1%–2% against CS-LSTM, while capturing lane-changing behavior and driver interaction successfully. The D-STGCN model (Sighencea et al., 2023) used spatio-temporal graph convolutions to predict pedestrian trajectories on ETH, UCY, and Stanford Drone datasets, providing ADE ≈ 0.42 m and FDE ≈ 0.68 m, beating existing methods with real-time performance based on positional information alone.

The SE-STDGNN model (Guo et al., 2025), based on EvolveGCNs with CNN and MLP layers, presented a self-evolving mechanism for online adaptation, and on the AD4CHE congested highway dataset, it enhanced prediction accuracy by 13% compared to CS-LSTM and Attention-LSTM, with real-time inference efficiency. A goal-oriented prediction model (Li et al., 2025) integrated a multi-head attention transformer for intention prediction with neural differential equations and a social force model for trajectory prediction, evaluated on NGSIM highways, that resulted in state-of-the-art long-term accuracy with improved interpretability. Finally, the HCAGCN framework (Lu et al., 2023) encoded vehicle dynamics, scene context, and changing interactions into heterogeneous graph convolutions, and on the INTERACTION and NGSIM datasets, it cut down ADE by 14.3% and FDE by 12.5% while maintaining real-time adaptability in complicated traffic scenarios.

Among the trajectory prediction models currently available, two significant limitations still exist. Firstly, a large majority of methods risk privacy by requiring vehicles to transmit over the network the raw data from sensors or trajectories during the training phase. Secondly, these methods face challenges when deployed across decentralized vehicular environments containing varying trajectory distributions. Hence, the overall prediction performance of such models becomes limited.

Unlike previous studies that employ STSDGNN in centralized settings, the proposed framework embeds the existing STSDGNN architecture within a federated learning environment for decentralized autonomous driving. Therefore, the novelty of this work lies not in proposing a new graph neural network architecture or federated optimization algorithm, but in designing and evaluating a unified privacy-preserving system that enables collaborative trajectory prediction under decentralized learning. Additionally, both GCN- and GAT-based spatial encoders are evaluated within the same federated framework to analyze their effectiveness.

The standard FedAvg algorithm is employed as a stable and widely adopted federated optimization strategy for aggregation of collaborative models between distributed clients.

## 2 Materials and methods

### 2.1 System description

The proposed system is developed to perform privacy-preserving vehicle trajectory prediction using a federated spatiotemporal deep learning framework. The system leverages distributed vehicle trajectory data to collaboratively learn motion patterns without sharing raw data, thereby addressing privacy, security, and data-ownership challenges in autonomous driving ecosystems. A vehicle trajectory datased *D* is denoted by Equation 1.


$$\begin{array}{l} D={\left\{T_{i}\right\}}_{i=1}^{N} \end{array}$$ (1)

where each trajectory Ti consists of a continuous sequence of spatial coordinates recorded over time *i* (1 to N). From this continuous stream, discrete training samples are generated using sliding window segmentation.

For each vehicle instance, an observation sequence and future prediction sequence are defined as:

- Observation window (*T*_*obs*_):
- A sequence of past positions $T_{obs}\in {\mathbb{R}}^{T_{obs}\times 2}$, where *T*_*obs*_ = 30 frames representing historical (*x, y*) coordinates.
- Prediction window (*T*_*fut*_):
- The corresponding future trajectory $T_{fut}\in {\mathbb{R}}^{T_{fut}\times 2}$, where *T*_*fut*_ = 10 frames, which serves as the ground-truth label.

Thus, each training sample is represented in Equation 2.


$$\begin{array}{l} S_{i}=\left(T_{obs},T_{fut}\right) \end{array}$$ (2)

To model the spatial structure, each sample is transformed into a graph representation as defined in Equation 3.


$$\begin{array}{l} G=\left(V,E,X,y\right) \end{array}$$ (3)

where:

- *V* denotes the set of nodes that represents vehicles.
- *E* denotes the edge set that captures spatial relationships between vehicles.
- *X* represents the embeddings of the node features.
- *y* denotes the prediction label (future trajectory).

In the graph representation, the graph will consist of the target vehicle as well as the other surrounding vehicles which are within the given spatial range. Because of this, the graph will always have multiple nodes to model the interactions by applying a Graph Neural Network. However, when the surrounding vehicles are not within the interaction range, the graph will be maintained with self-loop (|*V*| = 1).

The node feature tensor that represents the full observation history is defined as: *X* ∈ ℝ^(1 × 30 × 2)^.

The associated prediction label is stored as: *y* ∈ ℝ^(10 × 2)^.

#### 2.1.1 Spatiotemporal feature representation

The system learns motion dynamics through dual encoders that extract spatial and temporal embeddings.

- The spatial embedding is denoted as: $h_{s}\in {\mathbb{R}}^{64}$, capturing the current positional context of the vehicle.
- The temporal embedding is denoted as: $h_{t}\in {\mathbb{R}}^{64}$, summarizing the evolution of the historical motion.

These embeddings are synchronized via feature fusion to produce a unified latent representation, as shown in Equation 4.


$$\begin{array}{l} h_{c}=f_{sync}\left(h_{s},h_{t}\right) \end{array}$$ (4)

where $h_{c}\in {\mathbb{R}}^{64}$. This fused vector encodes the complete spatiotemporal state of the vehicle.

#### 2.1.2 Trajectory prediction representation

The decoder module maps the latent embedding to the future trajectory space, as defined in Equation 5.


$$\begin{array}{l} {\hat{T}}_{fut}=f_{pred}\left(h_{c}\right) \end{array}$$ (5)

where:

- ${\hat{T}}_{fut}\in {\mathbb{R}}^{\left(10\times 2\right)}$ represents the predicted future coordinates.
- pred_len = 10 denotes prediction horizon.

#### 2.1.3 Federated learning representation

To ensure privacy preservation, the proposed system adopts a federated learning framework called FedAvg, where multiple distributed clients collaboratively train a global model without sharing raw trajectory data.

Let:

- *K* denote the total number of clients
- *w*_*k*_ denote the local model parameters of client *k*

The global model parameters *W* are updated according to Equation 6.


$$\begin{array}{l} W=\frac{1}{K}\overset{K}{\underset{k=1}{\sum }}w_{k} \end{array}$$ (6)

In this setup, all clients contribute equally to the update of the global model. This ensures that every client participates uniformly in the aggregation process. This approach maintains simplicity in computation and allows efficient merging of models, making it a suitable choice for scalable federated learning frameworks.

Training proceeds over multiple communication rounds, where selected clients perform local optimization on their private trajectory graphs and transmit only updated model weights to the central server for aggregation.

### 2.2 System architecture

**Figure 1:**
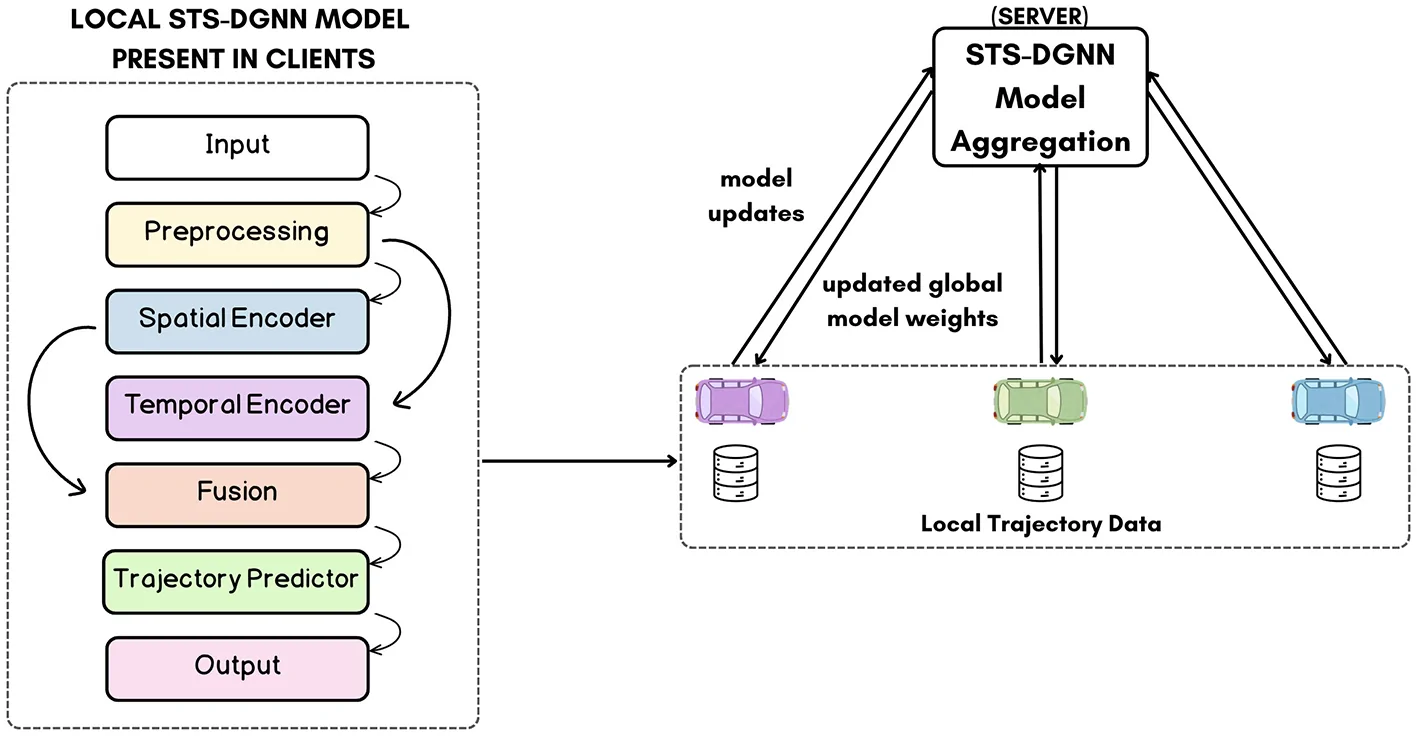
Federated learning setup for the STSDGNN model.

The architecture outlined in Figure 1 is an FL framework, combined with a STDGNN for trajectory prediction. This framework ensures the privacy-preserving training of a model, allowing various clients to collaboratively learn a shared model without sharing their private data. The system operates in a client-server topology to coordinate model updates and aggregation in a distributed client environment. The complete workflow can be separated into two principal components:

- Server Process - responsible for the initialization, distribution, and aggregation of the models.
- Client Process - performs local training using the private data while adjusting the model's weights.

The server and clients communicate in multiple federated rounds, allowing for iterative improvements in the model while keeping the data secure and private.

#### 2.2.1 Server side architecture

The server serves as the primary coordinator in the federated architecture. Its primary functions are model initialization, sharing, aggregation, and global updates.

##### 2.2.1.1 Data preparation and segmentation

The server first loads the raw dataset (*D*_*raw*_) containing trajectory data (e. g. 01_tracks.csv). The dataset is segmented into observation and prediction sequences using the given lengths (*T*_*obs*_ = 30, *T*_*pred*_ = 10).

Each observation sequence is transformed into a graph format, with nodes indicating points in space-time and edges indicating motion relations. The resultant data is separated into training and testing data (8:2) and shared among the N clients.

##### 2.2.1.2 Model initialization

The global STDGNN model is initialized with random parameters and the shared architecture is synchronously employed across clients. This model includes both spatial and temporal modules to extract trajectory dependencies.

##### 2.2.1.3 Client selection and model sharing

In every communication round (*R*), *K* clients are randomly chosen from the pool of clients. The server distributes the current global model weights to the selected clients for local training.

##### 2.2.1.4 Model aggregation

After the local training is complete, each client returns only updated model parameters and the server aggregates the collected weights using the federated averaging algorithm.

##### 2.2.1.5 Global Assessment and update

During the assessment's subsequent updates, the model is evaluated on a central test set related to convergence or progress being made on reducing test loss during each round. Working as independent clients (training nodes) in the network, each client has incomplete datasets for training (subset of the overall data) and works independently to preserve data privacy and local training performance.

#### 2.2.2 Client side architecture

##### 2.2.2.1 Local data

All clients have access to their data after it has been preprocessed and prepared as graphs with observation and predictive pairs. These data loaders (representing the number of clients in the network) are then capable of supporting mini-batch training.

##### 2.2.2.2 Local training

Each client initializes its model locally with the weights downloaded from the server. The local model is trained for *E* local epochs using Root Mean Squared Error (RMSE) as a loss function, for gradient-based optimization using the Adam optimizer. The Spatial Module encodes relationships/interactions to trajectory points, and the Temporal Module learns the trajectory transition over time when the model is being trained.

##### 2.2.2.3 Updating the client model

After finishing the individual client's local training, only the client's updated model weights are transmitted back to the server. Neither the raw data nor the sensitive data is shared between the clients and the server, protecting overall data security and maintaining the privacy of local data.

#### 2.2.3 Model architecture (STSDGNN)

The STSDGNN model is designed to model dynamic interactions in sequential trajectory data by combining graph based spatial reasoning and temporal sequence learning in an end to end manner.

##### 2.2.3.1 Input layer

Input features consist of observed trajectory points that exist as coordinates (*x*,*y*) over time-steps. The input data shape is of the form (*T*_*obs*_ × 2), where each point denotes spatial coordinates at a timestamp.

##### 2.2.3.2 Graph construction module

Each sequence of data is formatted as a spatio-temporal graph denoted by *G* = (*V*,*E*), where *V* = node (position) and *E* = edge representation of spatio-temporal dependencies. Edge connections are defined on the basis of time-step continuity or proximity between points.

This spatial neighborhood based graph construction guarantees the existence of the spatial relation only when the interactions exist for the specified condition. The constructed graph structure varies from sample to sample, even for multi-node interaction graphs to single-node graphs in low-density traffic conditions.

##### 2.2.3.3 Spatial encoder

The Graph Convolution Layer (GCL) is responsible for learning spatial dependencies among connected points by aggregating information among connected neighboring nodes that help inform the spatial structure and trajectory of motion.

##### 2.2.3.4 Temporal encoder

A Recurrent Neural Network (RNN) layer (e.g., LSTM, GRU) denotes the next layer; an RNN layer is representative of the temporal dynamics of motion. An RNN captures the evolution of an object's position over time, within the specific time sequence.

##### 2.2.3.5 Synchronization module

The outputs from the spatial- and temporal-encoders are subsequently fused together to produce a joint spatio-temporal embedding. This stage of the data synchronization process allows the model to understand both position and time dependencies simultaneously.

##### 2.2.3.6 Trajectory prediction(decoder) module

The combined embedding is forwarded through a fully connected decoder that provides the predicted future trajectory (*T*_*pred*_ × 2). The final output is the predicted (*x, y*) coordinates for future time steps.

### 2.3 Methodology

The proposed federated framework is evaluated against the original centralized STSDGNN implementation proposed by Li et al. (2023), which serves as the baseline model in this study. The baseline performs trajectory prediction using centralized training, where all trajectory data is aggregated and processed on a central server without federated optimization.

**Figure 2:**
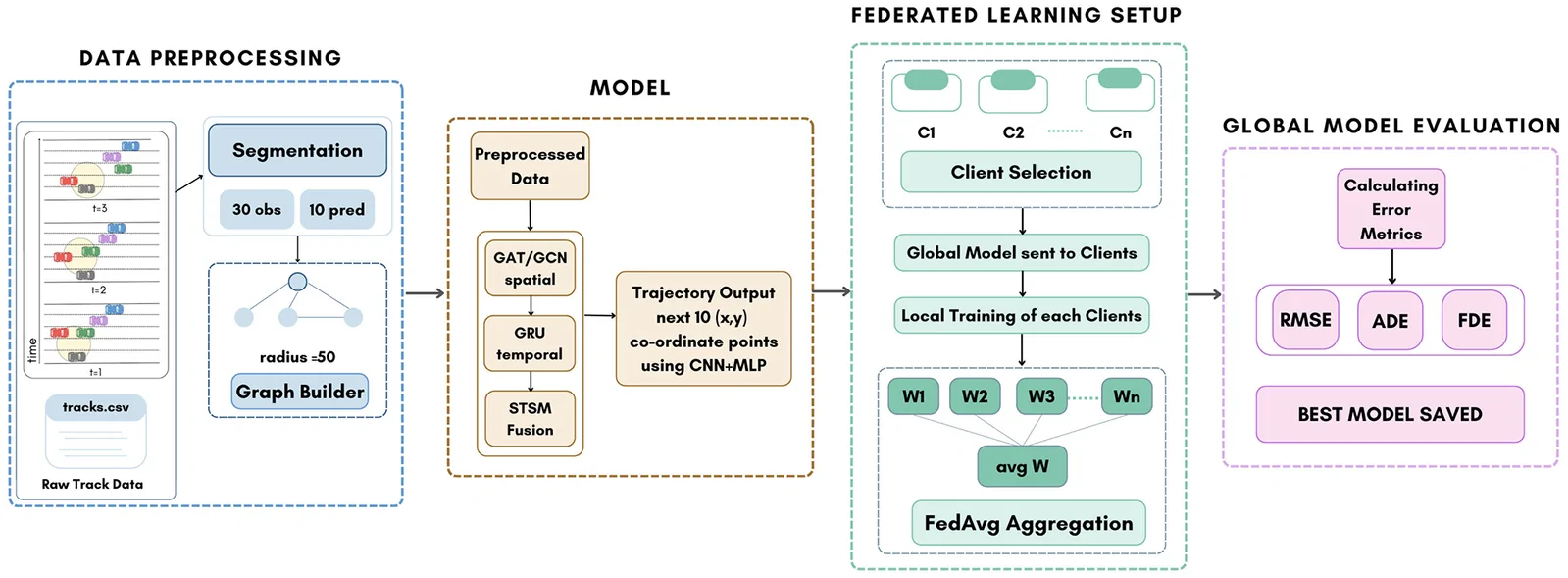
Detailed flow diagram.

The detailed flow of the implementation is shown in Figure 2.

#### 2.3.1 Experimental setup

The experimental setting adopted for federated training is presented in Table 1. All experiments used the same data processing pipeline, representation method for trajectories, observation time window, prediction time window, optimizer settings, and performance measures, thus providing an identical setting to compare the performance of both centralized and federated training setups. Training takes place with the use of the Adam optimizer with a learning rate of 0.001, a batch size of 16 and 3 training epochs per each communication round. During federated training, 10 communication rounds are performed. These hyperparameters were determined via preliminary experiments to provide for stable convergence.

**Table 1:** Training and model hyperparameters.

Parameter	Value
Number of rounds (*num*_*rounds*)	10
Local epochs (*local*_*epochs*)	3
Number of clients (*num*_*clients*)	10
Selected clients per round (*num*_*selected*)	5
Batch size (*batch*_*size*)	16
Patience (*patience*)	3
Sequence length (*seq*_*len*)	30
Prediction length (*pred*_*len*)	10
Interaction radius (*radius*)	50
Hidden dimension (*hidden*_*dim*)	64
Input dimension (*in*_*dim*)	2

#### 2.3.2 Data preprocessing module (DPM)

This module takes the continuous highD dataset (Krajewski et al., 2018) and creates graph-structured data for federated learning.

##### 2.3.2.1 Trajectory Data Preparation

The main objective is to change the continuous time series data in “*n*”_tracks.csv into discrete training samples. The function segment_sequence looks through each unique vehicle ID to apply a sliding window and create observation and future pairs (*T*_*obs*_, *T*_*fut*_), as defined earlier. A sequence of seq_len = 30 past (*x*,*y*) coordinates (*T*_*obs*_). This gives a historical context for the model. The associated sequence of pred_len = 10 future (*x*,*y*) coordinates (*T*_*fut*_). This will be the ground-truth label for the model to learn. Thus, each segmented sample forms an input-label pair used for supervised trajectory forecasting.

##### 2.3.2.2 Construction of trajectory graphs

Each pair of (Obs,Fut) is used to construct a graph object *G* = (*V*,*E*) via the build_graph function. In this formulation, the graph captures the target vehicle along with surrounding agents, where each node represents an individual vehicle. The node features encode the 30-frame observation history as tensors of shape (30,2), corresponding to the sequence of 2D positional coordinates over time.

Edges between nodes are established based on a spatial proximity criterion defined by a 50-unit radius in the build_graph function, enabling the modeling of local interactions between nearby vehicles. In cases where no neighboring agents fall within this radius, a self-referential edge configuration (i.e., edge_index = [[*i*],[*i*]]) is maintained to preserve compatibility with graph-based operations and ensure stable message passing.

The corresponding 10-frame future trajectory *T*_*fut*_ of the target vehicle is assigned as the prediction label for the graph, represented as a tensor g.y of shape (10,2).

##### 2.3.2.3 Spatial-temporal synchronization module (STSM)

This is the most important component of the STSDGNN model–multi-layer neural networks that pull and fuse features from the graph-structured input data. A spatial encoder and a fusion block provide a hierarchical structure.

###### 2.3.2.3.1 Spatial encoder

The spatial encoder is designed to capture the vehicle's spatial context at the current time step. It takes as input the node's trajectory feature sequence of shape (1, 30, 2), representing 30 time steps of 2-D positional coordinates, along with the graph connectivity information defined by the edge index. Instead of processing the entire sequence, the encoder extracts only the final position (i.e., the coordinates at *t* = 30, resulting in a tensor of shape (1, 2) that represents the most recent (*x*,*y*) location of the vehicle. This coordinate is then passed through a GCNConv layer to generate a higher-dimensional spatial representation. Mathematically, this operation is expressed in Equation 7.


$$\begin{array}{l} H=ÂXW \end{array}$$ (7)

where *X* denotes the feature matrix of the node, *W* represents the learnable weight matrix, and Â corresponds to the normalized adjacency matrix encoding the vehicle interaction relationships.

For the GAT variant, spatial representations are learned by assigning adaptive attention weights which are computed using Equation 8.


$$\begin{array}{l} \alpha_{ij}=\frac{\exp\left(\text{LeakyReLU}\left(a^{T}\left[Wx_{i}||Wx_{j}\right]\right)\right)}{\underset{k\in N\left(i\right)}{\sum }\exp\left(\text{LeakyReLU}\left(a^{T}\left[Wx_{i}||Wx_{k}\right]\right)\right)}, \end{array}$$ (8)

where α_*ij*_ denotes the normalized attention coefficient between nodes *i* and *j*, *a* is the learnable attention vector, N(i) represents the neighborhood of node *i*, and || denotes the concatenation operation.

In cases where there are only self-loop edges, the graph convolution essentially computes the output using features of the same node while still retaining the same formula for computing the result. For situations that involve interaction between several vehicles within a traffic scenario, the normalized adjacency matrix ensures that the encoder is able to perform feature aggregation from neighboring nodes to account for interactions.

The output obtained from the above operations is then mapped to a 64-dimensional spatial embedding *h*_*s*_.

###### 2.3.2.3.2 Temporal encoder

The temporal encoder captures the sequential motion dynamics of the target vehicle by modeling its historical trajectory. It takes as input the full observation sequence *T*_*obs*_, which represents the positional coordinates of the vehicle for the last 30-frames. This sequence is processed using a Gated Recurrent Unit (GRU), which is well-suited for learning temporal dependencies such as velocity changes, acceleration patterns, and turning behavior in time-series data. The GRU encodes the sequential information into a compact representation, and its final hidden state is extracted as the temporal embedding. This embedding is denoted as *h*_*t*_, which summarizes the overall motion dynamics of the vehicle.

###### 2.3.2.3.3 Synchronization block

The synchronization block integrates spatial and temporal representations to form a unified spatiotemporal embedding. Specifically, the spatial embedding *h*_*s*_, representing the current positional context, is combined with the temporal embedding *h*_*t*_, which captures the motion history. These two 64-dimensional vectors are concatenated to form a 128-dimensional feature vector. This combined representation is then passed through a fully connected layer (128 → 64), followed by a ReLU activation function to introduce non-linearity and enhance feature learning. The output of this block is a unified embedding *h*_*c*_, which encodes both the current spatial state and historical motion patterns of the vehicle.

#### 2.3.3 Trajectory prediction module (TPM)

The trajectory prediction module acts as a decoder that generates future trajectory estimates from the learned spatiotemporal embedding. It takes the unified representation *h*_*c*_ as input and transforms it into the future trajectory predicted over the defined prediction horizon. The embedding is first processed through a one-dimensional convolution layer (Conv1D), which serves as a feature mixing mechanism across the latent dimensions. This is followed by a global average pooling operation to reduce feature variability and produce a stable representation. Finally, a fully connected MLP layer maps the resulting 64-dimensional vector to the output space of size, (pred_len × 2). The output vector is then reshaped to obtain the predicted trajectory ${\hat{T}}_{fut}$, representing the future positions of the vehicle.

#### 2.3.4 Federated learning module (FPM)

This module describes the distributed training strategy used to optimize the STSDGNN model in a privacy-preserving manner. Instead of centralized training, the proposed framework adopts a FedAvg approach, enabling multiple clients to collaboratively learn a global model without sharing raw trajectory data.

##### 2.3.4.1 Client data partitioning

The complete dataset is initially divided into a global training set (80%) and a global test set (20%), where the test set is retained on the central server for the final evaluation. The training portion is further partitioned into **K** = 10 clients using a random splitting strategy, resulting in a largely balanced data distribution across clients. Although there may be slight differences in the nature of the data distribution in the local environment based on the diversity in the trajectory data sets, the general approach can be viewed as an approximate IID setup. Therefore, the current experimental setup represents a simplified federated learning scenario.

##### 2.3.4.2 Federated averaging (FedAvg) training

Training is conducted over multiple communication rounds (set to 10), involving iterative coordination between the central server and a subset of participating clients. In each round, a group of clients is randomly selected, and the current global model parameters are transmitted to them.

Each selected client performs local optimization on its private data using the Adam optimizer to minimize prediction loss. After local training, the updated model parameters are sent back to the server.

The server performs global model aggregation using the standard FedAvg algorithm, where the local model updates from the participating clients are averaged to produce the updated global model. The updated global model is then redistributed to clients for the next communication round. This iterative process continues until convergence, enabling the model to learn collaboratively while preserving data privacy.

## 3 Results

### 3.1 Dataset description

The highD dataset contains driving data from more than 110,500 vehicles, which represents one of the largest data collections of vehicle trajectories and has been utilized in research on vehicle trajectory prediction. Each highD vehicle trajectory has a number of different features, such as location or speed. The training and test dataset was created in a 8:2 split. In addition, the trajectories were segmented into segments of 40 frames. The first 30 frames of trajectory data were seen as historical input for each segment, while the last 10 frames of trajectory data were used as the label value. The dataset consisted of over 3 lakh sequences.

### 3.2 Evaluation metrics

RMSE is used as the primary metric for overall trajectory prediction accuracy comparison, while ADE and FDE evaluate the average trajectory deviation and the final predicted position accuracy, respectively.

Root mean square error (RMSE) calculates the square root of the average squared difference between each predicted trajectory point and its corresponding ground-truth coordinates, yielding an overall indication of prediction accuracy. RMSE exposes larger errors through the squaring process and can indicate the general reliability of the model estimates. In mathematical terms, RMSE is represented using Equation 9.


$$\begin{array}{l} \text{RMSE}=\sqrt{\frac{1}{T\;\cdot 2}\overset{T}{\underset{t=1}{\sum }}\left[{\left(x_{t}^{\text{pred}}-x_{t}^{\text{true}}\right)}^{2}+{\left(y_{t}^{\text{pred}}-y_{t}^{\text{true}}\right)}^{2}\right]} \end{array}$$ (9)

where *T* denotes the prediction horizon and (*x*_*t*_, *y*_*t*_) represents the spatial coordinates at time step *t*.

Average displacement error (ADE) invokes the mean Euclidean distance across all of the predicted positions and their corresponding actual position across the whole prediction time horizon. It indicates how well the model reliably follows the true trajectory from the start to the end of the prediction time series, and is represented using Equation 10.


$$\begin{array}{l} \text{ADE }=\frac{1}{T}\overset{T}{\underset{t=1}{\sum }}∥{\text{p}}_{t}^{pred}-{\text{p}}_{t}^{true}∥ \end{array}$$ (10)

where T is the prediction horizon, and *p*_*t*_ = (*x*_*t*_, *y*_*t*_) represents the 2D position at time step *t*.

Final displacement error (FDE) quantifies the Euclidean distance between the predicted final point and the ground-truth final point, which essentially indicates a model's accuracy in terms of long term prediction. FDE is particularly important in trajectory planning tasks, where the end position is the ultimate goal, and is expressed using Equation 11.


$$\begin{array}{l} \text{FDE }=∥{\text{p}}_{T}^{pred}-{\text{p}}_{T}^{true}∥ \end{array}$$ (11)

where *T* denotes the final prediction time step.

### 3.3 Quantitative results

Table 2 presents the performance of the proposed model using both GCN and GAT-based spatial encoder. The findings reveal that the GAT model is ahead of the GCN model across all evaluation metrics. Furthermore, the efficacy of GAT compared to GCN depends on the number of nodes available within the proximity created by the graph. This means that the more cars there are that interact in the environment, the better the spatial relationship modeling by the attention component of GAT. However, when we have only one node as a result of sparsity, there will be no effect due to added attention mechanism at all, and GAT works in the same way as GCN due to linearity. The results show that learning remains stable throughout several iterations of training, with measures such as RMSE, ADE, and FDE gradually converging. Reduced RMSE scores suggest that the trajectory prediction from a general perspective is more accurate, whereas the smaller ADE and FDE scores point to a closer fit to the actual trajectory not only for the whole prediction time, but also at the final time step.

**Table 2:** Performance comparison of GCN and GAT.

Model	RMSE	ADE	FDE
GCN	0.26	0.30	0.31
GAT	0.14	0.16	0.23

The improvement in FDE is particularly significant, since it indicates whether the model has really figured out where the vehicle will be at the end of that time period—something that is very important for real-life scenarios like avoiding one vehicle crashing into another or knowing what path to take.

### 3.4 Federated convergence behavior analysis

**Figure 3:**
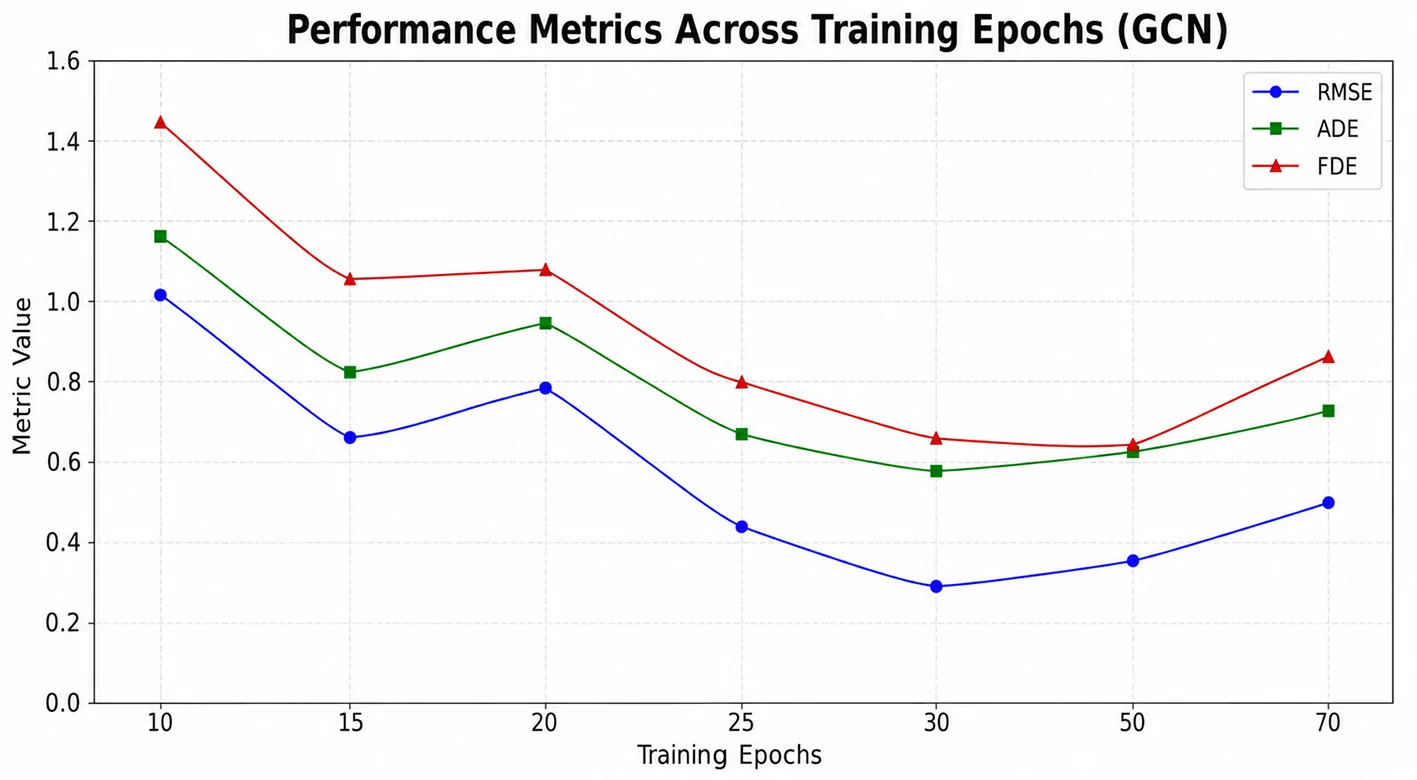
Performance metrics across training epochs (GCN).

**Figure 4:**
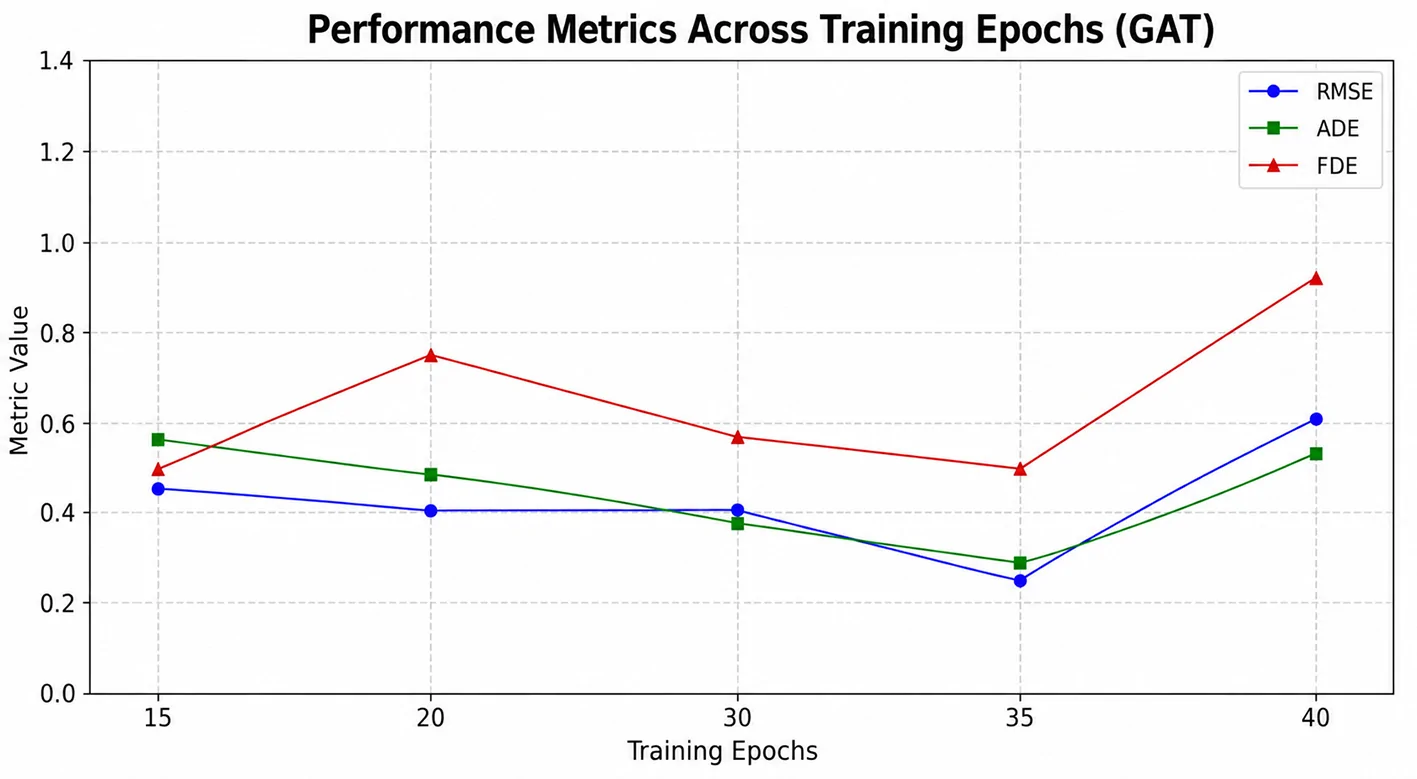
Performance metrics across training epochs (GAT).

Performance trends across different training epochs demonstrate stable optimization behavior during federated trajectory prediction training. The prediction error decreases progressively as the training epochs increase, with optimal performance observed around 30 epochs for the GCN encoder in Figure 3 and 35 epochs for the GAT encoder in Figure 4.

Beyond this point, performance degradation is observed for certain metrics, which may indicate the onset of overfitting during extended local training.

**Figure 5:**
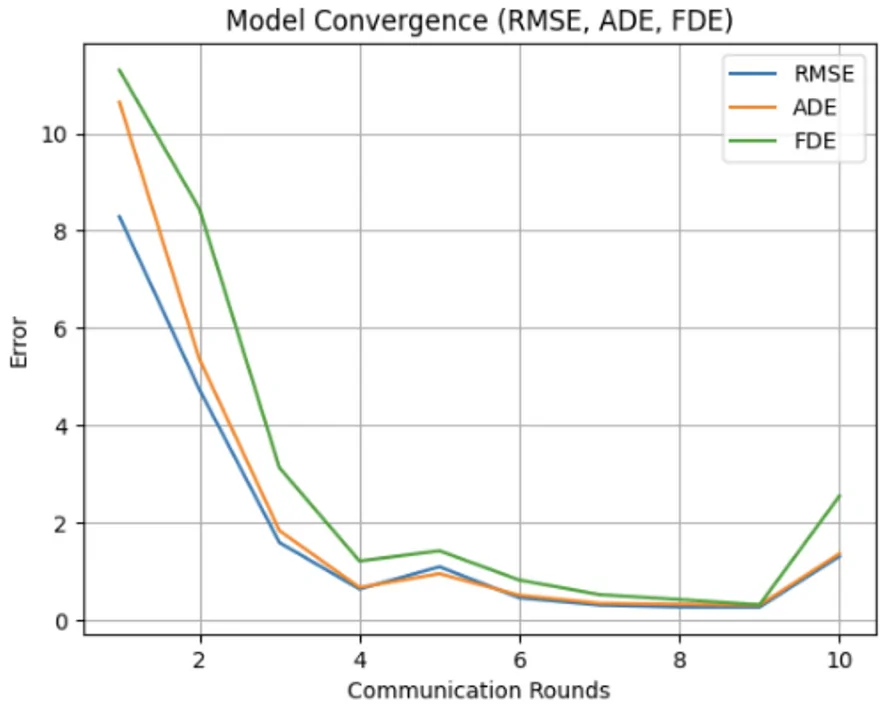
Federated convergence behavior of the GCN-based STSDGNN model across communication rounds.

**Figure 6:**
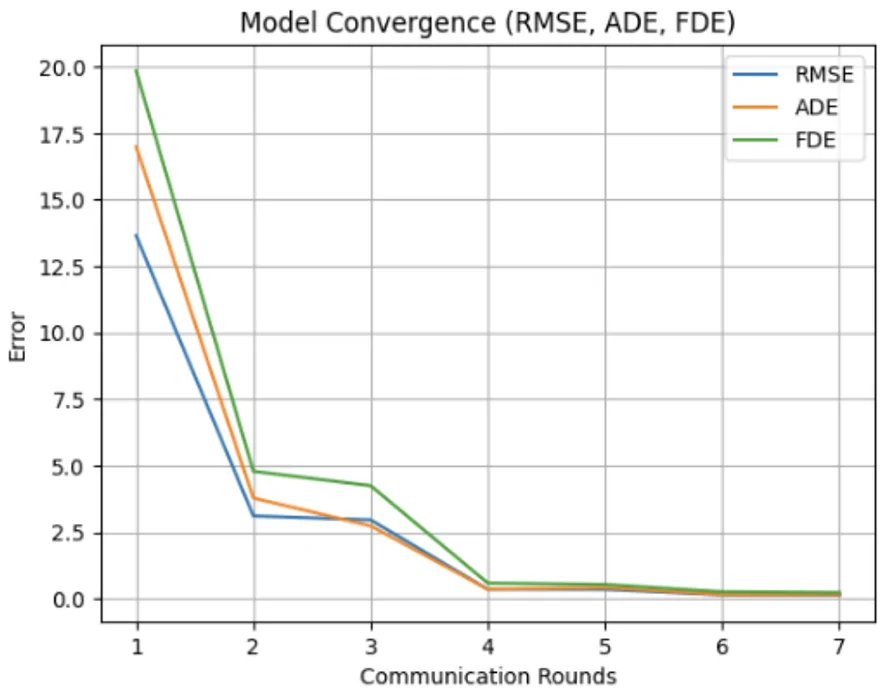
Federated convergence behavior of the GAT-based STSDGNN model across communication rounds.

Figures 5, 6 illustrate the convergence behavior and optimization stability of the federated training process across multiple communication rounds for the GCN and GAT-based models, respectively, under the best-performing epoch configuration.

Figure 5 depicts the convergence of the federated GCN-based STSDGNN model. The training loss is steadily decreasing as part of the optimization process, showing the stability of the learning algorithm even with decentralized updates on the client side and periodic aggregation at the server.

The steady decrease in the value of loss implies that the FedAvg aggregation procedure ensures coordination in the process of updating locally distributed models without any optimization instabilities. However, the speed of convergence proves to be somewhat slower than the one achieved in the case of the GAT-based model due to the uniform aggregation of neighboring vehicles used in GCN layers.

Figure 6 shows the convergence of the federated GAT-based STSDGNN model. As compared to the GCN-based model, the GAT-based model converges faster and attains a lower error in prediction.

The reason for such an advantage is the attention-based weighting of the neighborhood aggregation process, allowing for focusing on more informative spatial dependencies between neighboring vehicles when combining features.

### 3.5 Computational complexity analysis

#### 3.5.1 Time complexity

The computational complexity of the proposed Federated STSDGNN model involves local spatio-temporal graph learning independently at each participating client, followed by federated model aggregation through the FedAvg algorithm. Let *N* be the number of participating clients, *R* be the number of communication rounds, *E* be the number of local training epochs, *S* be the number of trajectories processed by each client, *T* be the trajectory sequence length, and *H* be the dimension of hidden features.

The local model training is done through the forward and backward propagation over its local trajectory sequences. Thus, the computational complexity for local model training would be approximately calculated using Equation 12.


$$\begin{array}{l} O\left(E\times S\times T\times H\right) \end{array}$$ (12)

Considering all the participating clients over R communication rounds, the overall computational complexity of the federated training process is given by Equation 13.


$$\begin{array}{l} O\left(R\times N\times E\times S\times T\times H\right) \end{array}$$ (13)

#### 3.5.2 Communication complexity

Communication overhead occurs as a result of the transmission of model parameters between the central server and participating clients at each communication round in federated learning. The total number of trainable model parameters is represented by *P*.

As participating clients download the global model and upload their own trained models per communication round, the communication complexity is represented by Equation 14.


$$\begin{array}{l} O\left(R\times N\times P\right) \end{array}$$ (14)

which increases linearly with both the number of communication rounds and the number of participating clients.

#### 3.5.3 Memory complexity

Every client maintains a local copy of the model parameters along with the feature representations computed locally during training. Thus, the memory complexity per participant is approximately given by Equation 15.


$$\begin{array}{l} O\left(P+B\times T\times H\right) \end{array}$$ (15)

where *B* denotes the batch size used during local training.

#### 3.5.4 Scalability analysis

The described federated approach allows for the implementation of distributed training on multiple participants, where the computation load is shared by local devices and only the model parameters are transmitted between the participant and the central server. As the number of participants increases, the communication load scales linearly since there are more parameters to be transferred during federated training. Likewise, an increase in the graph size or trajectory lengths leads to higher computational costs of local model training. However, the decentralized training scheme makes it possible for the approach to scale up.

### 3.6 Qualitative results

**Figure 7:**
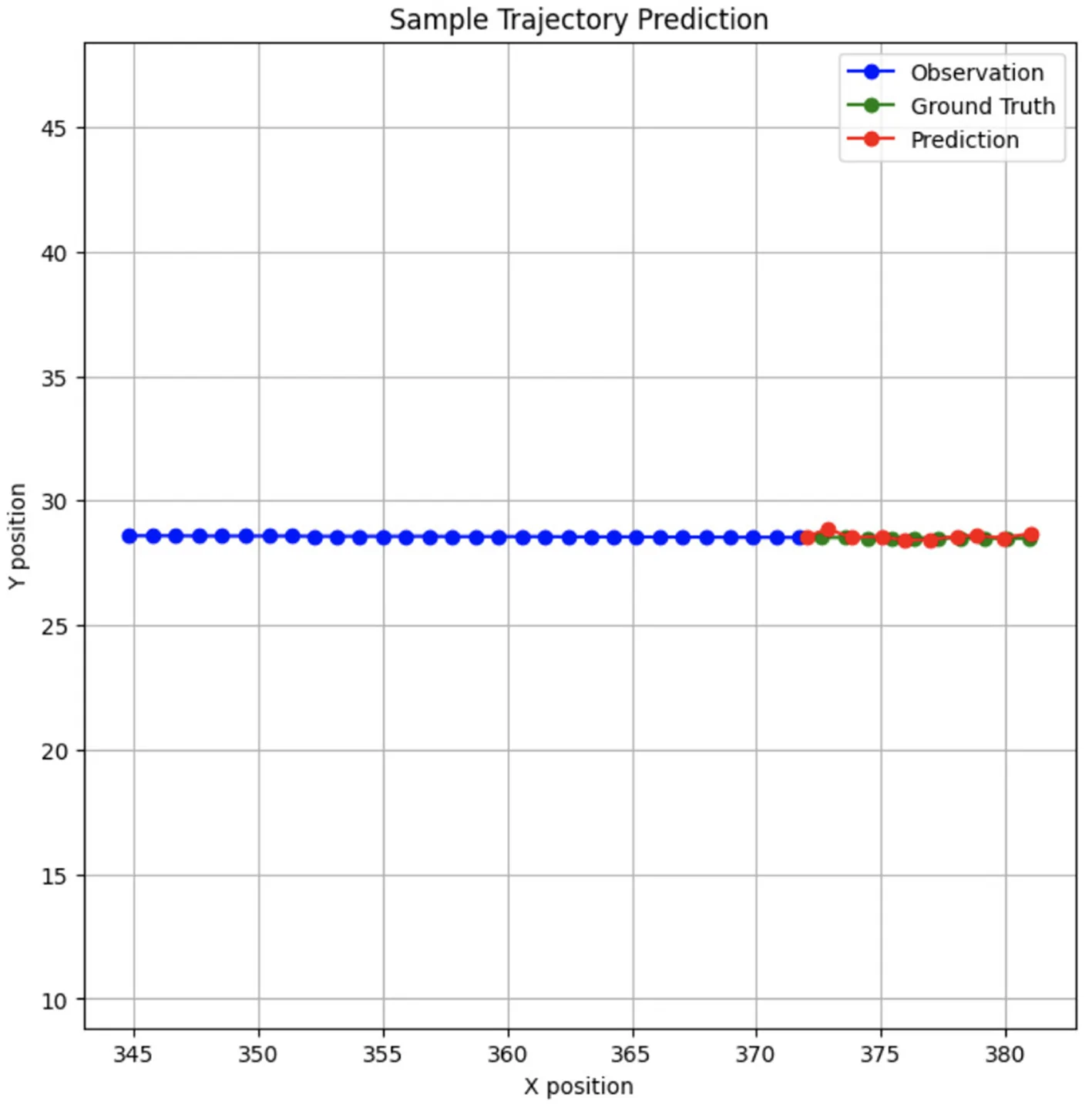
Sample trajectory prediction plot.

Figure 7 shows a sample trajectory prediction: the observed trajectory in blue, the ground-truth future trajectory in green, and the predicted trajectory in red. The observed trajectory shows an almost linear motion along the horizontal direction, which is consistent with constant velocity.

The predicted trajectory is very similar to the ground-truth one, which means that it captures direction and motion continuity efficiently. The difference between the predicted and actual positions is very minimal, indicating high accuracy in short-term predictions. Even at the end of the prediction period, the difference is quite small, which means that the model can produce reliable long-term forecasts. This is also confirmed by the low values of RMSE, ADE, and FDE in the quantitative evaluation.

### 3.7 Comparative analysis with baseline

A comparison was conducted between the proposed federated STSDGNN framework and the original centralized STSDGNN implementation proposed by Li et al. (2023) in order to evaluate the effect of federated decentralized training on trajectory prediction performance as seen in Table 3. Performance comparisons are reported using the evaluation metrics RMSE, ADE, and FDE. It is important to note that the comparison presented in Table 3 is conducted under differing experimental configurations, particularly with respect to the prediction horizon and the length of the input sequence. Due to these variations, a direct quantitative comparison between the proposed model and the baseline methods may not be strictly fair. Therefore, the comparative analysis presented in the work should be interpreted as a qualitative reference rather than a definitive quantitative benchmark against all other trajectory prediction methods. In the centralized scenario, the complete dataset is collected in one place for the model to run across the dataset. In the federated approach, the model is learning together with several clients, each holding their own partition of the dataset. The federated model matched the accuracy in the centralized model and also had higher precision scores across all metrics evaluated in the measures.

**Table 3:** Comparison between centralized STSDGNN baseline and proposed federated STSDGNN framework.

Model	RMSE	ADE	FDE
Baseline model	0.34 / 0.47	0.65 / 0.69	1.16 / 1.20
Proposed model	0.26 / 0.14	0.30 / 0.16	0.31 / 0.23

The performance improvement observed in the proposed federated framework is specific to the experimental configuration adopted in this study. Although the centralized and federated models were trained using the same overall dataset, differences in the training paradigm can influence the observed results. Therefore, improvement should not be interpreted as a universal advantage of federated learning. Nevertheless, within the experimental setting considered in this study, several factors may explain the observed performance gain. Federated learning allows collaborative training on a distributed, client-level dataset that may improve model generalization in decentralized learning systems. Furthermore, due to the increased input diversity of the training data, the model also benefits from an additional form of regularization in the representation learning of the spatial and temporal encoder modules. Additional repeated experimental trials and statistical significance analysis is required to further validate the consistency and reproducibility of these observations.

**Figure 8:**
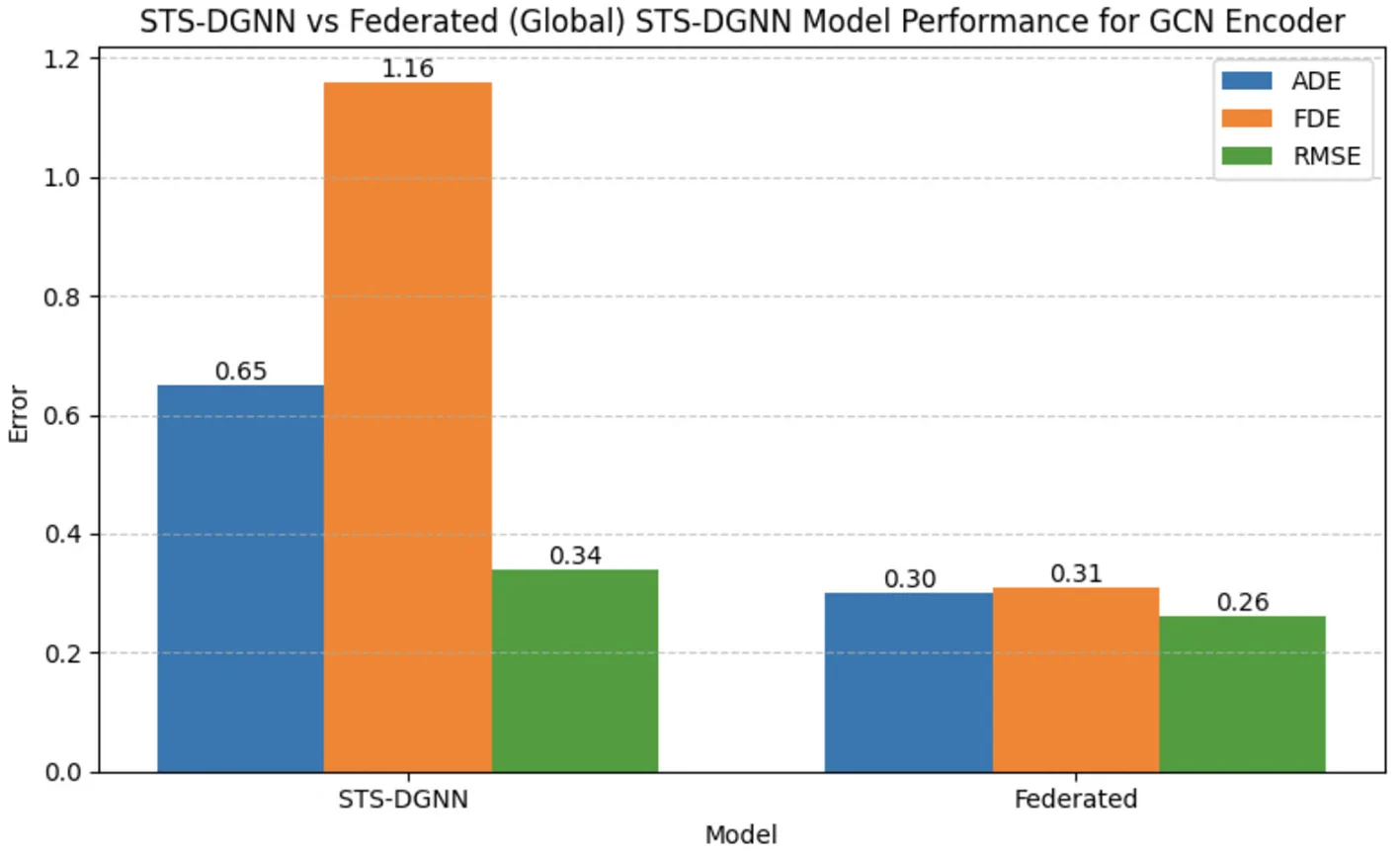
Comparison graph for GCN.

**Figure 9:**
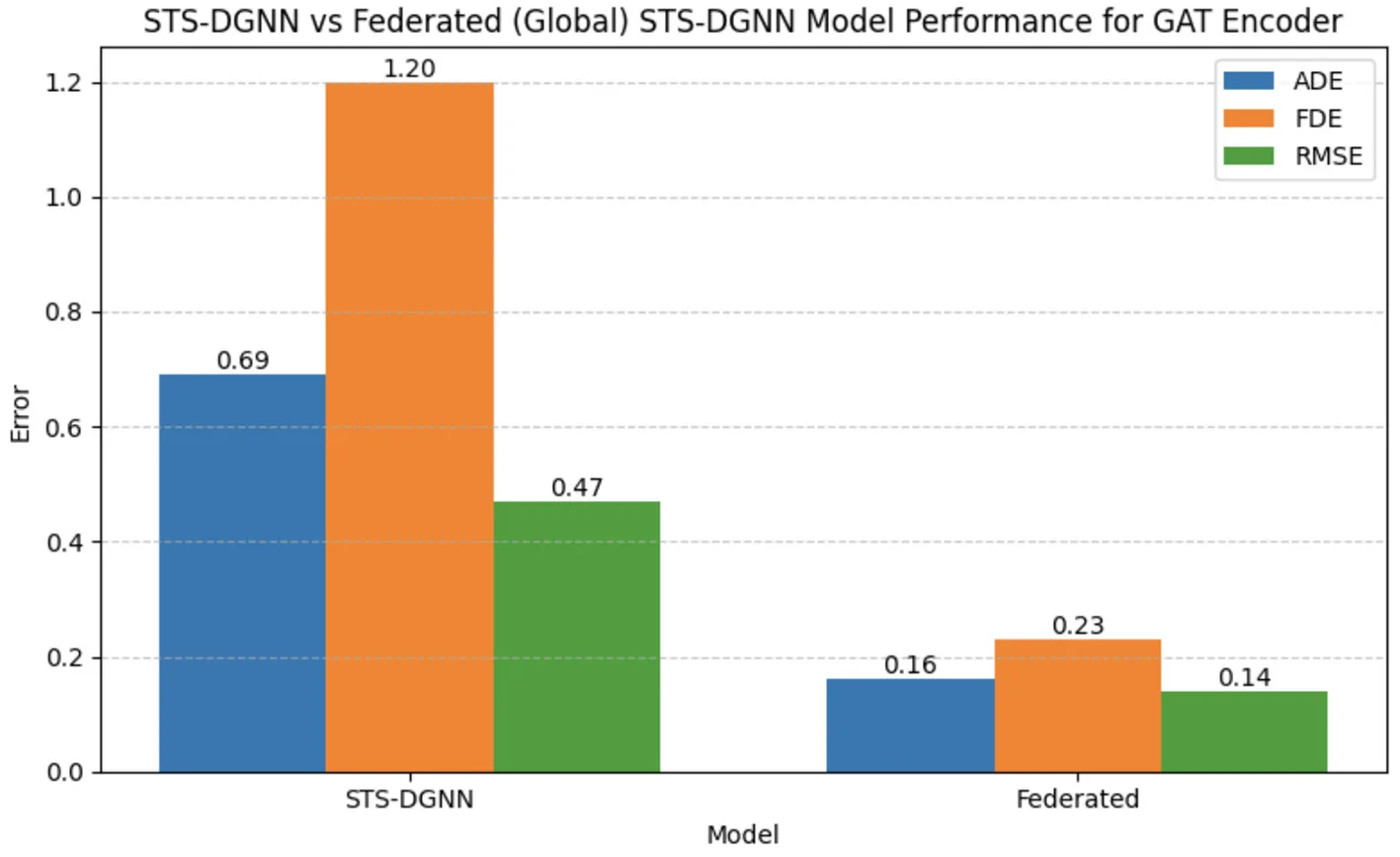
Comparison graph for GAT.

The comparison graphs in Figures 8, 9 also support this finding by illustrating the difference between the two models. Across all three metrics, the Federated Learning-STSDGNN curve is lower than that of the centralized model. This reinforces the finding that, in addition to providing data privacy, federated learning has overall improved model quality. The findings seem to challenge the notion that federated learning will often always show a reduction in accuracy compared to traditional learning due to client heterogeneity. The observed performance improvement should be interpreted only within the near-IID experimental setting considered in this study.

## 4 Discussion

The experimental results highlight several important observations.

GAT consistently outperforms GCN in every evaluation metric. The attention mechanism allows GAT to weigh spatial features by relevance. GCN applies equal weight to neighbors, missing subtle differences. That reduces its ability to capture fine spatial details. GAT's feature maps are more expressive. This leads to better trajectory predictions, hence increasing the model accuracy. The low ADE values show consistent accuracy across the prediction window. FDE values are also lower. This means that the final position is predicted more precisely. Motion planning and collision avoidance are based on this precision. It performs well both early and later in the forecast. There is no drop in performance as time passes.

The adoption of federated learning allows collaborative model training between multiple clients in a decentralized manner to protect data privacy. In the current model, the trajectory dataset is divided into different client partitions to mimic federated learning among vehicles without any centralized data exchange. Although client partitions are created randomly by splitting the dataset, there may be small discrepancies in the trajectory distribution between different clients due to the variation in trajectory fragments and vehicle trajectories. This indicates that the current experiment reflects the use of a federated learning system that is nearly independent and identically distributed or slightly heterogeneous, but not strongly heterogeneous. Federated aggregation allows the model to efficiently integrate information learned from diverse client datasets with stable convergence during communications. While federated learning outperformed centralized learning in the current experimental setup, more repeated experiments and formal significance tests are needed to establish the superiority of federated learning over centralized learning.

Although federated learning ensures that there is no exchange of the original trajectory data, privacy threats cannot be completely eliminated using federated learning alone. Gradient leakage and membership inference attacks are some of the privacy threats that can be leveraged by exchanging model parameters. Differential privacy, secure aggregation, and model aggregation using encryption are ways that can be used to enhance privacy in this case.

This study provides an evaluation of the proposed federated STSDGNN framework through an experimental setting, where the highD dataset is used for testing with almost IID distribution of clients. Although the proposed framework exhibits convergence and better trajectory prediction performance in this experimental setting, further experimental validation is required to establish its robustness across more diverse federated learning scenarios.

## 5 Conclusion

Autonomous driving research has shown that federated learning (FL) can support collaborative model training without the need to centralize raw data, thus ensuring privacy across distributed vehicle networks. However, a majority of current systems have only concentrated on communication optimization or general privacy mechanisms, and have mostly ignored the incorporation of dynamic spatial-temporal modeling for precise trajectory forecasting.

The main novelty of this work lies in the introduction of an existing STSDGNN in the context of federated learning in order to perform trajectory prediction in a privacy-preserving manner. Instead of developing a novel graph neural network or a federated learning method, this paper shows how the combination of a dynamic spatial-temporal graph model with a federated learning algorithm allows us to do trajectory prediction in a privacy-preserving manner.

While the proposed framework shows some encouraging results under the employed experimental setup, there are still some directions left to explore in future work. They include evaluation of the performance in realistic settings with non-IID client distribution using sophisticated federated optimization methods like FedProx and SCAFFOLD, privacy-preserving approaches, testing on other trajectory benchmarks, and more thorough experimental evaluation by multiple repetitions of experiments with statistical analysis and component-wise ablation studies.

## Data Availability

The original contributions presented in the study are included in the article/supplementary material, further inquiries can be directed to the corresponding author.
